# Myeloperoxidase and Eosinophil Peroxidase Inhibit Endotoxin Activity and Increase Mouse Survival in a Lipopolysaccharide Lethal Dose 90% Model

**DOI:** 10.1155/2019/4783018

**Published:** 2019-09-30

**Authors:** Robert C. Allen, Mary L. Henery, John C. Allen, Roger J. Hawks, Jackson T. Stephens

**Affiliations:** ^1^Department of Pathology, Creighton University School of Medicine, Omaha, NE 68124, USA; ^2^Exoxemis, Inc., Omaha, NE 68110, USA; ^3^Concord Biosciences, LLC, Concord, OH 44077, USA

## Abstract

Myeloperoxidase (MPO) and eosinophil peroxidase (EPO) are cationic haloperoxidases with potent microbicidal and detoxifying activities. MPO selectively binds to and kills some Gram-positive bacteria (GPB) and all Gram-negative bacteria (GNB) tested. GNB contain endotoxin, i.e., lipopolysaccharide (LPS) comprising a toxic lipid A component. The possibility that MPO and EPO bind and inhibit the endotoxin of GNB was tested by mixing MPO or EPO with LPS or lipid A and measuring for inhibition of endotoxin activity using the chromogenic Limulus amebocyte lysate (LAL) assay. The endotoxin-inhibiting activities of MPO and EPO were also tested *in vivo* using an LPS 90% lethal dose (LD90) mouse model studied over a five-day period. Mixing MPO or EPO with a fixed quantity of LPS from *Escherichia coli* O55:B5 or with diphosphoryl lipid A from *E. coli* F583 inhibited LAL endotoxin activity in proportion to the natural log of the MPO or EPO concentration. MPO and EPO enzymatic activities were not required for inhibition, and MPO haloperoxidase action did not increase endotoxin inhibition. Both MPO and EPO increased mouse survival in the LPS LD90 model. In conclusion, MPO and EPO nonenzymatically inhibited *in vitro* endotoxin activity using the LAL assay, and MPO and high-dose EPO significantly increased mouse survival in a LPS LD90 model, and such survival was increased in a dose-dependent manner.

## 1. Introduction

Myeloperoxidase (MPO) is a unique dimeric heme A glycoprotein produced by neutrophil and monocyte leukocytes [1, 2]. Eosinophil leukocytes produce a monomeric eosinophil peroxidase (EPO) with some (72.4% nucleotide and 69.8% amino acid) homology to MPO [3–5]. Both MPO and EPO are cationic, but EPO is more cationic than MPO [6]. Both enzymes show classical peroxidase and haloperoxidase (XPO) activities. MPO and EPO catalyze the oxidation of chloride and bromide, respectively. The haloperoxidase activities of both enzymes are highly microbicidal [7, 8]. MPO production in neutrophils is normally abundant [9] and is increased by treatment with recombinant granulocyte colony-stimulating factor (rhG-CSF) and by host inflammation [10]. In addition to microbe killing, the haloperoxidase activity of MPO is reported to inactivate microbial toxins, including diphtheria toxin, tetanus toxin, and *Clostridium difficile* cytotoxin [11–13]. As with microbicidal action, the toxin-destroying activities of MPO require haloperoxidase action and are hydrogen peroxide (H_2_O_2_) and halide dependent.

Gram-negative bacteria (GNB) are composed of an outer cell wall membrane presenting LPS anchored by its toxic lipid A component [14]. MPO selectively binds to GNB and kills many Gram-positive bacteria (GPB) and all GNB tested [15]. The MPO binding observed for all GNB tested suggested the possibility that endotoxin, a characteristic component of GNB [16–18], might be involved in such binding. Direct MPO binding and/or inhibition of the endotoxin activity of lipopolysaccharide (LPS) or lipid A has not been reported. However, MPO-deficient mice show increased mortality in a polymicrobial sepsis model [19], and more recently, Reber et al. have reported that blockade or genetic deletion of MPO significantly increased mortality associated with LPS challenge [20].

## 2. Materials and Methods

### 2.1. Enzymes

The purified haloperoxidases, porcine myeloperoxidase (MPO) and porcine eosinophil peroxidase (EPO), were produced by Exoxemis, Inc. No recombinant enzymes were used in the research described herein. Dilutions of MPO, EPO, and MPO-GO stock solutions for *in vitro* and *in vivo* testing were made with low endotoxin reagent water (LRW).

The porcine MPO used was 98.9% pure by ultraperformance liquid chromatography (RP-UPLC) and 100% pure by molecular size exclusion high-performance liquid chromatography (SEC-HPLC). The sodium dodecyl sulfate-polyacrylamide gel electrophoresis (SDS-PAGE) results for porcine MPO conformed with the MPO standard. MPO had a *A*_430nm_/*A*_280nm_ Reinheitszahl (RZ) of 0.79. The guaiacol unit (GU) activity of the porcine MPO was 404 GU/mg; 1.0 GU of activity consumes 1.0 *μ*mol H_2_O_2_/minute [21]. The suspension medium for the stock 6 mg/mL MPO was 50 mM acetate buffer containing 100 mEq/L NaCl plus 0.01% Tween-80 at a pH of 5.3.

The porcine EPO used was 99.2% pure by reversed-phase high-performance liquid chromatography and had a *A*_415nm_/*A*_280nm_ RZ of 0.96. The SDS-PAGE results for porcine EPO conformed with standard EPO. The guaiacol unit (GU) activity of the porcine EPO was 80 GU/mg [21]. The suspension medium for the stock 14.2 mg/mL EPO was 20 mM sodium phosphate buffer containing 150 mM NaCl plus 0.01% Tween-80 with a pH 6.0.

Glucose oxidase (GO) was isolated from *Aspergillus niger* and purified to 99.8% by RP-HPLC and 99.9% by SEC-HPLC by Exoxemis, Inc. The unit (U) activity of GO was 309 U/mg; 1.0 U oxidizes 1.0 *μ*mol of *β*-D-glucose to D-gluconolactone and H_2_O_2_/minute at pH 5.1 at 35°C [22]. The GO suspension medium was 200 mM potassium phosphate buffer with a pH of 7.0.

### 2.2. Endotoxins

Lipopolysaccharide (LPS) purified from *E. coli* O55:B5 was purchased from Sigma-Aldrich (L4524) with a specified LPS activity of 3 × 10^6^ endotoxin units (EU) per mg. This activity was consistent with our chromogenic Limulus amebocyte lysate assay results. Diphosphoryl lipid A purified from *E. coli* F583 (Rd mutant) was purchased from Sigma-Aldrich (L5399) with a specified lipid A activity of 1 × 10^6^ EU per mg. Our LAL assay, run in an aqueous milieu without organic solvents, showed much lower activity, but this lower activity was consistent in multiple runs. Hydrophobic interaction is most likely responsible for the relative loss of available lipid A surface for interaction with LAL and XPO. Our assay results were reproducible, but the EU values do not quantitatively reflect the mass of lipid A tested. Without the use of a solubilizing agent, the LAL measurements are only qualitative, but testing was performed to determine if MPO and EPO could qualitatively inhibit the endotoxin activity of lipid A.

### 2.3. Chromogenic Limulus Amebocyte Lysate Assay

The Limulus amebocyte lysate (LAL) reagent, prepared from amebocytes of the horseshoe crab, *Limulus polyphemus*, was used to assay the endotoxin activity of LPS and lipid A [23–25]. The chromogenic microplate LAL assay (LAL Endochrome-K, Endosafe) was purchased from Charles River. Endotoxin activation of the LAL clotting enzyme was quantified by measuring the enzymatic cleavage of a chromogenic substrate releasing p-nitroaniline (pNA). Activity was measured as the change in absorbance at a wavelength of 405 nm in a microplate spectrophotometer (Tecan). Kinetic measurement of the time-to-maximum color change was used to gauge the activity of endotoxin present. The time limit for detection was set at 1680 seconds (28 min). Calibration standards were prepared, and a standard curve was used to generate an equation with coefficient of determination (*R*^2^) for each experiment performed.

### 2.4. Chromogenic LAL Inhibition Testing

The chromogenic LAL method was adapted as an *in vitro* assay for measuring XPO inhibition of endotoxin. This inhibition assay included a preincubation step where either LPS or lipid A was mixed with the test enzyme (MPO or EPO) for a period of 30 minutes at 37°C. All test reagents and enzymes were diluted in low endotoxin reagent water (LRW). Following incubation, LAL solution was added and chromogenic activity was measured as the time (in seconds) to maximum color change. Inhibition was calculated as the difference between the endotoxin activity expected in the absence of XPO relative to the actual endotoxin activity of LPS or lipid A measured in the presence of MPO or EPO. Inhibition was expressed as a percentage of the endotoxin activity of LPS or lipid A alone.

MS Excel and IBM SPSS 17.0 software were used for data analysis, curve fitting, calculating the coefficient of determination (*R*^2^), adjusted *R*^2^ and standard error of the regression (*S*), ANOVA, and *F* statistic. The *R*^2^ measures the fit of the measured observations in proportion to total variation of outcomes predicted using the empirically generated equation, i.e., the proportion of variance in the dependent variable predictable from the independent variable [26]. The better the equation-data fit, the higher the *R*^2^ and the lower the *S*. The adjusted *R*^2^ and the standard error of the regression (*S*) are unbiased estimators that correct for the sample size. The adjusted *R*^2^ should be only slightly smaller than *R*^2^. Low adjusted *R*^2^ values indicate that insufficiently informative variables are fitted to an inadequate sample of data. The *F* statistic provides information as to the statistical significance of *R*^2^ model; the lower the *p*, the greater the significance.

### 2.5. Mouse LPS Ninety Percent Lethal Dose (LD90) Model

Experimentally naïve, healthy BALB/c female mice with a weight range of 16.2 to 19.7 g were divided into dose groups. Treatment of the animals (including but not limited to all husbandry, housing, environmental, and feeding conditions) was conducted in accordance with the guidelines recommended in *Guide for the Care and Use of Laboratory Animals*. All mouse testing was performed according to the protocols and standard operational procedures of Concord Biosciences, LLC, Concord, OH 44077.

Except for one group containing 15 mice, all test groups contained 20 mice. For all groups, each mouse was intraperitoneal (IP) injected with the indicated dose of purified LPS in a 0.5 mL total volume. LRW was used to adjust the concentrations of LPS. Groups 1, 2, 3, and 4 received 0.20, 0.35, 0.50, and 0.65 mg of LPS per mouse, respectively. After 5 days of observation, the censored (live) and the event (dead) mouse counts were tabulated. Group 1 had 15 live with 5 dead for a 25% lethality, group 2 had 5 live with 15 dead for a 75% lethality, and groups 3 and 4 had 20 dead with 0 live for 100% lethality. The 90% lethal dose (LD90) was estimated to be 0.40 mg per mouse, i.e., about 22 mg/kg.

The mouse LD90 testing was performed in two phases. The first phase tested the action of 0.5 mg MPO combined with the 0.4 mg LPS LD90 dose. The second phase expanded testing to include doses of 2.5 and 5.0 mg MPO in combination with the 0.4 mg LPS LD90 dose and also included doses of 2.5 and 5.0 mg EPO in combination with the 0.4 mg LPS LD90 dose. Two additional control groups received either 5.0 mg MPO or 5.0 mg EPO without LPS. Adjustments of the concentrations of LPS, MPO, and EPO were made using low endotoxin reagent water (LRW). The appropriate concentrations of LPS plus XPO were vortexed vigorously for about a minute then incubated at 37°C for 45 min. The mix was vortexed again prior to IP injection of 0.5 mL per mouse. Survivor analysis was performed with IBM SPSS software using the Laerd Statistics guide for Kaplan-Meier survival analysis. The log rank (Mantel-Cox) [27], Breslow (generalized Wilcoxon) [28], and Tarone-Ware [29] methods were applied for analysis and pairwise comparison. Statistical difference was considered significant when the probability (*p*) value was less than 0.05.

## 3. Results

### 3.1. Chromogenic LAL Assay Measurement of MPO and EPO Inhibition of LPS

**Figure 1:**
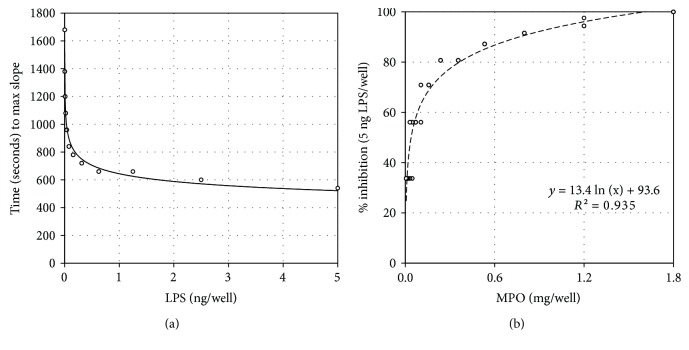
(a) Regression plot of LPS standards expressed as time (sec) to maximum slope against mass of LPS (independent variable). For the range: 0.0012-5 ng lipopolysaccharide (LPS)/well the derived equation was *y* = 643.96*x*^−0.131^ with a coefficient of determination (*R*^2^) of 0.982; for the range: 0.0012-0.625 ng LPS/well the equation was *y* = 600.99*x*^−0.146^ with an *R*^2^ = 0.991. (b) Plots the percent (%) inhibition of the Limulus amebocyte lysate (LAL) activity for 5.0 ng LPS/well (the dependent variable, *y*) against varying concentrations (mg/well) of myeloperoxidase (the independent variable, *x*). Testing was run in quadruplicate with all 60 data points depicted. The regression equation shows inhibition proportional to the natural log of the MPO mass with the *R*^2^ of 0.9348. Additional statistical information is described in Table 1.

Endotoxin-activated LAL cleavage of chromogenic substrate was measured spectrophotometrically, and the temporal kinetic endpoint of the time-to-maximum slope was used to quantify the endotoxin activity [23]. Calibration standards were run, and a standard curve with equation was generated with ng LPS/well plotted against the time point of maximum color change (max slope) as shown in Figure 1(a). This lot of LPS had an activity of 3 × 10^6^ endotoxin units (EU) per mg, i.e., 3 EU/ng. As expected, the 0.01 ng LPS/well standard had a time to max slope of 1200 sec, i.e., equivalent to about 0.03 EU. In Figure 1(b), % inhibition of the endotoxin activity for a fixed mass of LPS is plotted against MPO concentration.

The relationship of the independent variable (*x*), i.e., MPO mass (mg/well), to the dependent variable (*y*), i.e., % inhibition of a fixed mass of LPS (5 ng/well; 15 EU/well), is defined by the equation: *y* (% inhibition of 5 ng LPS) = 13.44 ln*x* (mg MPO) + 93.6, where Ln indicates that *x* is the natural log of the MPO mass. The strength of the relationship is indicated by the *R*^2^ value of 0.9348. Additional statistical analyses are presented in Table 1. The adjusted *R*^2^ and the standard error of the regression (*S*) are unbiased estimators that correct for the sample size and numbers of coefficients estimated. *S* is an absolute measure of the typical distance that the data points fall from the regression line in the units of the dependent variable, and provides a numeric assessment of how well the equation fits the data. The fact that the adjusted *R*^2^ value of 0.9337 is only slightly less than the *R*^2^ indicates that sufficiently informative variables are fitted to an adequate sample of data. The *F* statistic provides information for judgment as to whether the relationship is statistically significant, i.e., whether *R*^2^ is significant. A *p* < 10^−35^ is unquestionably significant.

**Figure 2:**
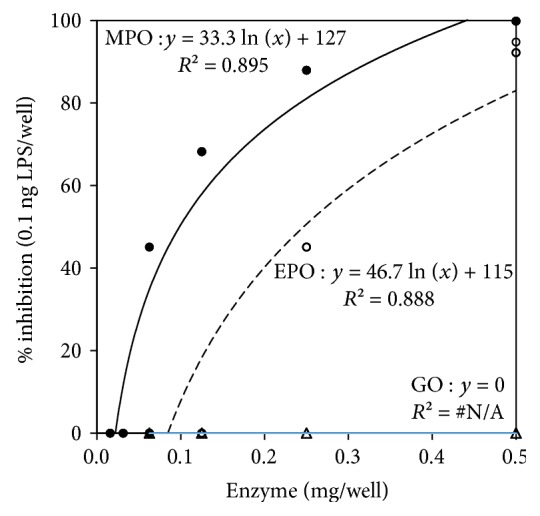
Percent inhibition of Limulus amebocyte lysate (LAL) endotoxin activity for 0.1 ng lipopolysaccharide (LPS)/well plotted against mg/well of myeloperoxidase (MPO), shown as filled circles, eosinophil peroxidase (EPO), shown as open circles, or glucose oxidase (GO), shown as open triangles (at 0 baseline; no gradient). The regression equations plus *R*^2^ values are shown for MPO and for EPO. The % inhibition of 0.1 ng LPS is proportional to the natural log of the MPO or EPO mass. GO produced no inhibition. Additional statistical information is described in Table 1.

Figure 2 depicts the plot of percent inhibition of a fixed 0.1 ng of LPS (dependent variable, *y*) against varying quantities of MPO, EPO, and GO (independent variable, *x*). Both MPO and EPO inhibited LPS, but MPO inhibition was superior to EPO. For a constant mass of LPS, inhibition of endotoxin activity is proportional to the natural log of the MPO or EPO mass. GO was included as a non-XPO control and did not inhibit endotoxin activity at any concentration tested.

### 3.2. MPO and EPO Inhibition of Lipid A Endotoxin Activity by the Chromogenic LAL

**Figure 3:**
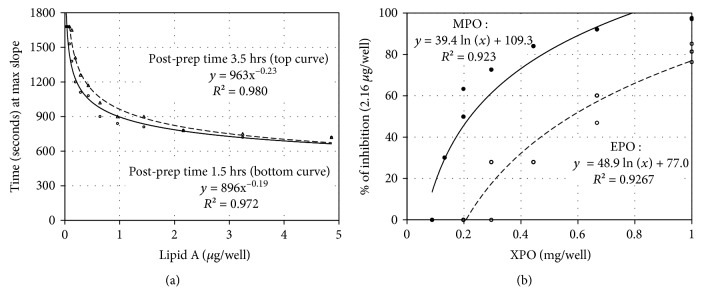
(a) Regression plot of lipid A standards (*µ*g/well) against time (sec) to maximum slope with derived equations and *R*^2^ values. The top and bottom curves were obtained from the same set of standards 1.5- and 3.5-hour postpreparation of the lipid A standards as indicated. (b) Percent inhibition of Limulus amebocyte lysate (LAL) activity for 2.16 *μ*g lipid A/well plotted against varying concentrations of MPO (filled circles) or EPO (open circles). The regression equations with *R*^2^ values for MPO and EPO inhibition of lipid A are shown. Lipid A standards were simultaneously run for each MPO or EPO inhibition experiment. Additional statistical information is described in Table 1.

Lipid A, the toxic component of LPS [16], has two glucosamine units with anionic phosphate groups attached and typically six hydrophobic fatty acids that anchor it into the outer membrane of GNB. Purified lipid A has endotoxin activity measurable with the LAL assay. The inhibitory actions of MPO and EPO were measured against the endotoxin activity of lipid A using the previously described chromogenic LAL assay. The results of lipid A standards tested over time are presented in Figure 3(a).

The hydrophobic character of lipid A complicates LAL quantification in an aqueous milieu [14], and as such, the measured endotoxin unit activity per lipid A mass is much lower than the manufacture reported value (i.e., ≥1 × 10^6^ EU/mg). Such LAL measurements are reproducible, but are qualitative. As illustrated by the data of Figure 3(a), lipid A endotoxin activity measured using the chromogenic LAL assay was reasonably stable over several hours. Using the equation generated from simultaneously run lipid A standards, the results of MPO and EPO inhibition of lipid A endotoxin activities are shown in Figure 3(b). MPO and EPO inhibited the endotoxin activity of lipid A, and such inhibition is proportional to the natural log of the MPO or EPO concentration. As observed for LPS, MPO is more inhibitory than EPO.

### 3.3. Haloperoxidase Enzymatic Action Is Not Required for Endotoxin Inhibition

**Figure 4:**
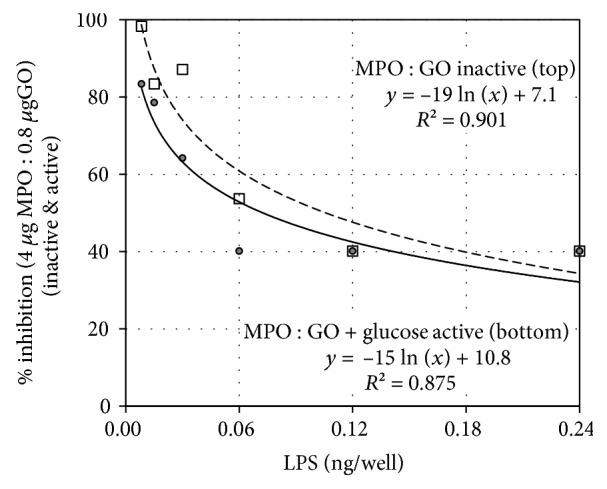
Percent inhibition of lipopolysaccharide (LPS) endotoxin activity for a formulation composed of 4 *μ*g myeloperoxidase (MPO) and 0.8 *μ*g glucose oxidase (GO) with 23 *μ*g Cl^−^/well measured against varying concentrations of LPS using the Limulus amebocyte lysate (LAL) assay. The formulation was tested without D-glucose, i.e., no haloperoxidase activity (indicated by white boxes), and with a nonlimiting concentration (0.2 mg/well) of D-glucose, i.e., haloperoxidase activity (indicated by the black dots). The data points at 0.12 and 0.24 LPS show overlap and appear as black boxes. The regression equations with *R*^2^ values show that the percentage inhibition of LPS endotoxin activity for a constant concentration of MPO plus GO is proportional to the negative log of the LPS concentration with or without D-glucose. Additional statistical information is described in Table 1.

The microbicidal and reported antitoxin activities of MPO require functional haloperoxidase activity [7, 13]. The experiments described in Figures 1–3 were conducted in the absence of H_2_O_2_ or a H_2_O_2_-generating system and, as such, did not involve enzymatic action. The experiment described in Figure 4 was designed to measure any difference in endotoxin inhibition related to haloperoxidase activity. The formulation contained 0.8 *μ*g GO as the H_2_O_2_-generating enzyme and 4.0 *μ*g MPO as the haloperoxidase. This MPO-GO formulation was tested in the absence of D-glucose and in the presence of D-glucose, with inhibition of LPS endotoxin activity measured by the LAL assay. The microbicidal action of this fully active MPO-GO-glucose formulation, i.e., E-101, has been reported [15, 30, 31].

With GO-MPO held constant and LPS varied, the percent inhibition of endotoxin activity is proportional to the negative natural log of the LPS concentration as depicted in Figure 4 and described in Table 1. As the LPS concentration increases, the ratio of MPO-to-LPS decreases. When MPO is held constant, percent inhibition is inversely related to the LPS (independent variable) present. With a nonrate limiting concentration of D-glucose as substrate and chloride as cofactor, the MPO-GO complex shows haloperoxidase activity [31]. However, both the enzymatically active and the enzymatically inactive MPO-GO complex inhibited LPS endotoxin activity with the inactive complex showing slightly higher inhibition. Haloperoxidase activity is not required for and does not improve endotoxin inhibition.

### 3.4. Effect of MPO and EPO on Survival in an Endotoxin LD90 Mouse Model

Intraperitoneal (IP) injection of 0.4 mg LPS (in a 0.5 mL total volume) per mouse produced about 90% lethality (LD90) in naïve, healthy BALB/c female mice. This LD90 dose is equivalent to about 22 mg/kg and was used for all LD90 testing. The indicated concentrations of LPS and MPO were mixed for about a minute, incubated at 37°C for 45 min, then vortexed again prior to IP injection (in a 0.5 mL total volume per mouse). All injected materials were treated in like manner. No H_2_O_2_ or H_2_O_2_-producing oxidase was present.

**Figure 5:**
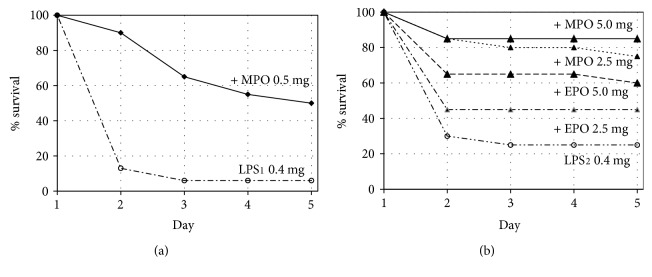
(a) Initial study of the effect of 0.5 mg myeloperoxidase (MPO)/mouse on survival depicted as Kaplan-Meier survivor curves for naïve BALB/c female mice over a 5-day period following IP injection of an 90% lethal dose (LD90) (0.4 mg lipopolysaccharide (LPS)/mouse; i.e., about 22 mg/kg) dose on day 1. (b) Follow-up study showing Kaplan-Meier survivor curves using 2.5 and 5.0 mg MPO/mouse and also 2.5 and 5.0 mg eosinophil peroxidase (EPO)/mouse.

The initial phase of testing measured the effect of a 0.5 mg MPO/mouse on the survivability of mice treated with the LD90 dose of 0.4 mg LPS/mouse. As depicted in the Kaplan-Meier plot of Figure 5(a) and described in the statistical analyses of Table 2, this 0.5 mg dose of MPO resulted in 50% lethality, a significant improved survival in this LPS LD90 model. Clinical observations for the MPO-treated group of mice were similar to those of the LPS-only group, but LPS-associated mortality was significantly decreased for the MPO-treated group.

The second phase of LD90 testing expanded the concentration range of MPO and included EPO for comparison of XPO effectiveness. The 0.4 mg LPS-only LD90 group for the second study showed less than the expected mortality, i.e., 75% instead of 90% mortality, but as described in the pairwise comparison of Table 2, log rank (Mantel-Cox) analysis of both the initial phase and second phase 0.4 mg LPS LD90 results shows a chi-square of 2.248 for a significance (*p* value) of 0.134, i.e., no significant difference [27]. Pairwise analyses of the both 0.4 mg LPS (LPS 0.4_1 and LPS 0.4_2) LD90 results by the Breslow (generalized Wilcoxon) [28] and the Tarone-Ware [29] methods also show lack of a statistically significance yielded *p* values of 0.098 and 0.106, respectively.

An MPO-only control group of twenty mice was treated with 5 mg MPO/mouse without LPS and showed no abnormal clinical observations and no mortality. Animals treated with 5 mg EPO without LPS also showed no abnormal clinical observations, but two animals were found dead in their cages the day following dosing for a lethality of 10%.

As depicted in Figure 5(b) and described in Table 2, increasing the concentrations of MPO to 2.5 and 5.0 mg/mouse significantly improved mouse survival in this 0.4 mg LPS LD90 model, and survival was increased in a dose-dependent manner. Pairwise comparison of the results described in Table 2 describes the significance of any group compared to any other group. Three different approaches were used for chi-square and *p* value. Comparison of the untreated 0.4 mg LPS LD90 mice with the 0.4 mg LPS mice treated with 2.5 and 5.0 mg MPO showed significances (*p* values) of 0.001 and 0.0001 using the log rank (Mandel-Cox) method, respectively. The *p* values were lower using the Breslow (generalized Wilcoxon) and the Tarone-Ware methods.

The 0.4 mg LPS groups treated with 2.5 and 5.0 mg EPO also showed improved survival in this 0.4 mg LPS LD90 model, and survival was increased in a dose-dependent manner as depicted in Figure 5(b). As described in Table 2 using the more stringent log rank (Mantel-Cox) method, pairwise comparison of the 0.4 mg LPS-only group with the 2.5 mg and 5.0 mg EPO treated 0.4 mg LPS groups shows *p* values of 0.1965 (not significant) and 0.0024 (significant), respectively.

## 4. Discussion

Strong MPO binding has been observed with many GPB, but members of the lactic acid family of GPB show relatively weak MPO binding [15, 32]. MPO binds with all GNB tested. The strength of MPO binding is proportional to the efficiency of haloperoxidase-mediated microbicidal action. When two bacteria are tested in combination and the concentration of MPO is limiting, i.e., a strong MPO-binding GNB with weak MPO-binding H_2_O_2_-producing viridans streptococci, microbicidal action is restricted to the GNB with sparing of streptococci. The primary and secondary microbicidal products of MPO action, i.e., hypochlorite and singlet molecular oxygen with its microsecond lifetime, favor the selective killing of MPO-bound microbes [15, 33, 34].

MPO inactivates microbial toxins including diphtheria toxin, tetanus toxin, and *Clostridium difficile* cytotoxin [11–13]. Like microbicidal action, these toxin-destroying activities of MPO required haloperoxidase enzymatic action. MPO binding and inhibition of endotoxin have not been described, but binding of MPO to all GNB tested suggests the possibility that such binding involves membrane endotoxin [15]. GNB have an outer cell wall membrane comprising LPS with its toxic lipid A component [14]. Even following bacterial death, endotoxin released can cause septic shock.

In addition to improving the potency and selectivity of microbicidal action, MPO and EPO binding to GNB appear capable of providing secondary protection against endotoxin. MPO and EPO inhibit the endotoxin activities of LPS and lipid A measured using the chromogenic LAL assay. Electrostatic interaction may play a role in cationic XPO binding to the anionic phosphate groups of LPS and lipid A, but electrostatic binding alone does not explain the approximate threefold greater inhibitory action of MPO relative to more cationic EPO on a mass basis. Contact is required for MPO and EPO inhibition of LPS and lipid A endotoxin activities, but endotoxin inhibition does not require haloperoxidase enzymatic activity. The results presented in Figures 1–3 were obtained in the absence of H_2_O_2_, and as such, the XPO were incapable of haloperoxidase action. When a GO-MPO formulation was tested for endotoxin inhibition in the presence and absence of H_2_O_2_ generation, as described in Figure 4, both the enzymically functional and nonfunctional haloperoxidase systems inhibited LPS endotoxin activity with the nonfunctional system showing slightly greater inhibition.

LAL is remarkably sensitive with regard to detection of LPS and lipid A. The observation that relatively high concentrations of MPO or EPO are required to demonstrate inhibition of the endotoxin activity using the LAL assay may reflect higher avidity of the LAL reagent for LPS and lipid A. High concentrations of MPO and EPO may be necessary for successful competition with the LAL reagent. Despite sensitivity limitations, MPO and EPO inhibition of LPS and lipid A endotoxin activities have been reproducibly demonstrated using the LAL assay. The % inhibition of endotoxin activity for a fixed quantity of LPS is proportional to the positive natural log of the quantity of XPO tested as described in Table 1.

As demonstrated by the results of Figure 4, enzymatically inactive MPO plus GO (without glucose) and active MPO plus GO (with glucose) formulations are roughly equivalent with respect to inhibition of LPS. Enzymatic activity is not required and does not improve MPO inhibition of LPS endotoxin activity. With the % inhibition of endotoxin for a fixed concentration of MPO as the dependent variable and the concentration of LPS as the independent variable, % inhibition is proportional to the negative natural log of the quantity of LPS test as described in Table 1.

The *in vitro* observations of MPO and EPO inhibition of endotoxin in the absence of enzymatic function suggested that MPO and EPO might provide innate protection against the pathophysiology of endotoxin shock. This possibility was explored using an *in vivo* mouse LPS lethal dose 90% (LD90) model to test if treatment with MPO or EPO could provide a survival advantage over untreated LD90 mice. As described in Table 2, treatment with 0.5 mg MPO/mouse, roughly equivalent to LD90 dose of 0.4 mg LPS/mouse, significantly increased mouse survival. Significant *in vivo* protection is achieved with an MPO-to-LPS mass inhibition ratio of 1.25. Treatment with higher doses of MPO further increased survival in a dose-dependent manner. EPO also significantly increases mouse survival, but only at the higher (5 mg EPO/mouse) dose. IP injection of 5 mg MPO/mouse (~275 mg/kg) without LPS caused no morbidity and no mortality. IP injection of 5 mg EPO/mouse without LPS caused no morbidity, but 10% lethality was observed.

## 5. Conclusion

All Gram-negative bacteria tested show MPO binding. Selective MPO microbial binding physically focuses its potent but transient oxygenation activities to the bound microbe for maximum microbicidal action with minimal host damage [15]. The *in vitro* and *in vivo* research results reported herein demonstrate that MPO, and to a lesser extent EPO, inhibits endotoxin activity. Unlike the microbicidal actions of XPOs, endotoxin inhibition does not require haloperoxidase enzymatic action. In addition to potent haloperoxidase microbicidal action, the evidence supports the conclusion that MPO and EPO also provide nonenzymatic protection against endotoxin. Inhibition of endotoxin using the *in vitro* LAL assay appears to require XPO binding and inactivation of LPS. Increased survival in the *in vivo* LD90 mouse model may also involve contact inactivation. This assumption is reasonable based on available data, but alternative mechanisms are possible and cannot be excluded. XPOs might compete or otherwise interfere with some *in vivo* mechanism(s) of endotoxin activation of inflammation. In the mouse LD90 studies, the non-LPS control group dosed with 5 mg MPO/mouse showed no morbidity and no mortality. MPO haloperoxidase action is potent but restricted. Such action is pH-restricted and H_2_O_2_-dependent. MPO and high-dose EPO protected against the LPS endotoxicity as demonstrated by increased survival in a LD90 mouse model. MPO-deficient mice have been reported to show increased mortality in a polymicrobial sepsis model [19], and more recently, blockade or genetic deletion of MPO has been reported to significantly increase mortality after LPS challenge [20]. The present observations demonstrate that MPO and EPO nonenzymatically inhibit endotoxin measured *in vitro* by the LAL assay, and increase *in vivo* survival in the mouse LD90 LPS model are consistent with these observations and support a role for MPO in innate protection against endotoxin.

## Figures and Tables

**Table 1:** Tabulated inhibition statistics for in vitro LAL assay studies.

Figures	Dependent variable, *y*	Independent variable, *x*	*y* = *β*_0_ + *β*_1_ Ln*x*		Coefficient of determination, *R*^2^	Adjusted *R*^2^	Std. error of the regression, *S*	*F* statistic change	Degrees of freedom		Sig. *F* change
			*β*0	*β*1					df1	df2	*p* value
1(b)	% inhibition (5 ng LPS)	MPO (mg)	93.6	13.4	0.9348	0.9337	6.34	831.7	1	58	4.4*E* − 36
2	% inhibition (0.1 ng LPS)	MPO (mg)	127.0	33.3	0.8945	0.8897	14.15	186.5	1	22	3.2*E* − 12
2	% inhibition (0.1 ng LPS)	EPO (mg)	115.0	46.7	0.8894	0.8815	13.65	112.6	1	14	4.5*E* − 08
2	% inhibition (0.1 ng LPS)	GO (mg)	N/A	N/A	N/A	N/A	N/A	N/A	N/A	N/A	N/A
3(b)	% of inhibition (2.16 *μ*g lipid A)	MPO (mg)	109.3	39.4	0.9233	0.9204	9.55	313.1	1	26	5.1*E* − 16
3(b)	% of inhibition (2.16 *μ*g lipid A)	EPO (mg)	77.0	48.9	0.9268	0.9228	8.29	228.0	1	18	1.2*E* − 11
4	% inhibition (4 *μ*g MPO: 0.8 *μ*g GO)	LPS (ng)	7.1	-19.1	0.9005	0.8756	9.03	36.2	1	4	0.0038
	Without glucose (inactive)										
4	% inhibition (4 *μ*g MPO: 0.8 *μ*g GO)	LPS (ng)	10.8	-15.0	0.8747	0.8434	8.04	27.9	1	4	0.0061
	With glucose (active)										

ANOVA and regression analyses were performed with IBM SPSS Statistics Software version 17.0.

**Table 2:** Tabulated Kaplan-Meier statistics for in vivo mouse LPS LD90 survival studies.

Pairwise comparisons														
Intervention (treatment)	LPS 0.4_1		LPS 0.4_2		LPS+MPO 0.5_3		LPS+MPO 2.5_4		LPS+MPO 5.0_5		LPS+EPO 2.5_6		LPS+EPO 5.0_7	
	Chi-square	Sig.	Chi-square	Sig.	Chi-square	Sig.	Chi-square	Sig.	Chi-square	Sig.	Chi-square	Sig.	Chi-square	Sig.
Log rank (Mantel-Cox)														
**LPS 0.4_1**			**2.25**	**0.133783**	**17.14**	**0.000035**	18.84	0.000014	20.45	0.000006	6.00	0.014344	11.12	0.000853
**LPS 0.4_2**	**2.25**	**0.13378**			**5.45**	**0.019534**	**10.78**	**0.001025**	**14.42**	**0.000146**	**1.67**	**0.196523**	**5.13**	**0.023508**
LPS MPO 0.5_3	17.14	0.00003	5.45	0.019534			2.19	0.139232	4.47	0.034591	0.63	0.427931	0.12	0.727124
LPS MPO 2.5_4	18.84	0.00001	10.78	0.001025	2.19	0.139232			0.55	0.457017	3.89	0.048615	1.05	0.304518
LPS MPO 5.0_5	20.45	0.00001	14.42	0.000146	4.47	0.034591	0.55	0.457017			6.86	0.008829	3.00	0.083262
LPS EPO 2.5_6	6.00	0.01434	1.67	0.196523	0.63	0.427931	3.89	0.048615	6.86	0.008829			0.91	0.340018
LPS EPO 5.0_7	11.12	0.00085	5.13	0.023508	0.12	0.727124	1.05	0.304518	3.00	0.083262	0.91	0.340018		
Breslow (generalized Wilcoxon)														
**LPS 0.4_1**			**2.74**	**0.097654**	**21.01**	**0.000005**	19.86	0.000008	20.45	0.000006	6.00	0.014344	11.61	0.000655
**LPS 0.4_2**	**2.74**	**0.09765**			**9.81**	**0.001732**	**11.92**	**0.000555**	**13.66**	**0.000219**	**1.22**	**0.269219**	**5.17**	**0.022957**
LPS MPO 0.5_3	21.01	0.00000	9.81	0.001732			1.72	0.189593	3.43	0.063996	2.53	0.111582	0.01	0.916648
LPS MPO 2.5_4	19.86	0.00001	11.92	0.000555	1.72	0.189593			0.41	0.520491	5.00	0.025303	1.32	0.249758
LPS MPO 5.0_5	20.45	0.00001	13.66	0.000219	3.43	0.063996	0.41	0.520491			6.86	0.008829	2.81	0.093939
LPS EPO 2.5_6	6.00	0.01434	1.22	0.269219	2.53	0.111582	5.00	0.025303	6.86	0.008829			1.21	0.272046
LPS EPO 5.0_7	11.61	0.00065	5.17	0.022957	0.01	0.916648	1.32	0.249758	2.81	0.093939	1.21	0.272046		
Tarone-Ware														
**LPS 0.4_1**			**2.610**	**0.106196**	**19.57**	**0.000010**	19.49	0.000010	20.45	0.000006	6.00	0.014344	11.45	0.000715
**LPS 0.4_2**	**2.61**	**0.10620**			**7.83**	**0.005128**	**11.51**	**0.000693**	**14.03**	**0.000180**	**1.40**	**0.237000**	**5.21**	**0.022470**
LPS MPO 0.5_3	19.57	0.00001	7.83	0.005128			1.96	0.161793	3.94	0.047104	1.49	0.221961	0.01	0.903650
LPS MPO 2.5_4	19.49	0.00001	11.51	0.000693	1.96	0.161793			0.48	0.488655	4.50	0.033908	1.20	0.274135
LPS MPO 5.0_5	20.45	0.00001	14.03	0.000180	3.94	0.047104	0.48	0.488655			6.86	0.008829	2.90	0.088505
LPS EPO 2.5_6	6.00	0.01434	1.40	0.237000	1.49	0.221961	4.50	0.033908	6.86	0.008829			1.08	0.299255
LPS EPO 5.0_7	11.45	0.00072	5.21	0.022470	0.01	0.903650	1.20	0.274135	2.90	0.088505	1.08	0.299255		

Analyses were performed with IBM SPSS Statistics Software version 17.0 using the Laerd Statistics guide for Kaplan-Meier survival analysis.

## Data Availability

Detailed data regarding the mouse lethal dose 90% (LD90) studies are available on request.
